# Improving Corrosion Resistance of Aluminosilicate Refractories towards Molten Al-Mg Alloy Using Non-Wetting Additives: A Short Review

**DOI:** 10.3390/ma13184078

**Published:** 2020-09-14

**Authors:** Faranak Barandehfard, James Aluha, AliReza Hekmat-Ardakan, François Gitzhofer

**Affiliations:** 1 Department of Chemical & Biotechnological Engineering, Université de Sherbrooke, Sherbrooke, QC J1K 2R1, Canada; faranak.barandehfard@usherbrooke.ca (F.B.); james.aluha@usherbrooke.ca (J.A.); 2 Research & Development Center, Pyrotek Inc., Sherbrooke, QC J1L 1W9, Canada; alihek@pyrotek.com

**Keywords:** aluminosilicate refractory, molten aluminum-magnesium, Alcan immersion test, mullite, non-wetting, corrosion

## Abstract

The corrosion of refractories in contact with high temperature aluminum-magnesium alloys leads to contamination of the Al-Mg alloy products by solid impurities from degraded refractories. Where both the spinel and corundum phases form in the refractories, cracks are generated and propagated by diffusion of molten Al-Mg, resulting in severe corrosion. In this review paper, the corrosion phenomenon is discussed, and published work is summarized, supplemented by our recent experimental results. Using the Alcan immersion test, materials based on white-fused mullite (WFM) were evaluated for their corrosion resistance and interfacial behavior. WFM was modified using different 2-wt.% of non-wetting additives (NWAs), such as BaSO_4_, CaF_2_, Secar^®^71 cement and wollastonite to improve their performance when in contact with molten Al-Mg alloy at 850 °C for 96 h. The mechanical properties of the samples such as flexural and compressive strength were evaluated, in addition to X-ray diffraction and microscopic analysis (optical and scanning electron microscopy coupled with X-ray elemental mapping). It was observed that cracks formed in samples were promoted with only BaSO_4_, CaF_2_, Secar^®^71 cement or wollastonite. However, cracks did not appear in the sample promoted with both 1-wt.% CaF_2_ and 1-wt.% BaSO_4_, because of improved anti-wetting properties in addition to inhibiting spinel (MgAl_2_O_4_) formation, which is the main cause of the cracks. This is a significant finding in the prevention of cracks and improvement of the refractory corrosion resistance.

## 1. Introduction

Aluminum and its alloys have unique properties such as very high strength-to-weight ratio, exhibiting perfect elasticity, superior malleability, easy machining ability, excellent corrosion resistance, good thermal and electrical conductivity, and it can be recycled or used repeatedly without any change in its properties. These outstanding characteristics of Al and its alloys promote the extensive use of the materials in various industries. Such applications involve infrastructure development and transportation machines or equipment. Consequently, the demand for Al production continues to increase annually, with the global market growing by about 5% year-on-year [1]. Since the Al industry has an annual turnover of about 60 million metric tons (MMT/year) in the world and represents about 90 billion USD, this demonstrates its importance in the world economy. Consequently, the consumption of refractories for molten Al alloy furnaces is also increasing dramatically [2]. Some of the biggest concerns in the Al industry touch on the environmental footprint and energy consumption, as the International Aluminum Institute has reported [3]. For example, producing one ton of Al requires 400 kg of carbon, which is a substantial amount. In addition, depending on the technology used and the age of the smelters, the energy consumption varies between 12.8–16 MWh for every ton of Al produced. Some issues of concern being addressed by the Al industry are greenhouse gas emissions [4,5,6], high energy demand [7,8], loss of aluminum through oxidation [9], recycling of Al scraps [10], corrosion of cathode lining [6], and corrosion of the refractory used in Al melting furnaces [11]. For example, Rio Tinto and Alcoa Corporation initiated a revolutionary Al manufacturing process through the Elysis project that produces oxygen and eliminates all direct greenhouse gas (GHG) emissions from the traditional smelting process. In order to reduce GHG emissions, carbon anodes can be replaced by inert anodes, as is the case in the Canadian Elysis project [12,13]. In China, lithium is used in the Al electrolysis process to decrease the operational temperature of the smelters, in order to lower the energy consumption [14]. Given the high erosion rate of graphitized cathodes, modified copper-insert collector bars and graphitic cathodes are used [15,16]. Some non-wetting additives (NWA) are incorporated in refractories that regularly come in contact with molten Al alloys to diminish their susceptibility to corrosion [17].

The low density displayed by Al and its alloys with excellent mechanical, thermal and electrical properties [18] provides a combination of unique properties that make Al and its alloys some of the most versatile and economically valuable metallic materials. The aluminum-magnesium (Al-Mg) series of alloys integrate lightweight and high strength characteristics with corrosion resistance properties. These properties lend the alloys extensive application in marine or seawater operations, construction of bulk road, ship structures, and chemical plants, where the alloys are exposed to robust corrosive environments [19]. Since Mg is a chemically active element and is eminently suitable in various types of reactions, its presence in molten Al enhances the reactivity of the Al-Mg alloy [20]. Some authors [21] have shown that when Mg as a highly reducing alloying element is added to molten Al, it exposes the furnace refractories to the aggressive and corrosive conditions of the molten alloys. The presence of Mg has two effects: (i) it reacts with aluminosilicate refractories to form magnesium aluminate spinel (MgAl_2_O_4_), which causes an expansion that consequently creates cracks and spalling in the refractories; and (ii) Mg lessens the viscosity of the molten alloy, and hence, it increases the penetration of the molten alloy into the porous structure of the refractory [22]. Therefore, frequent replacement of the refractories is required due to the constant physical and chemical damages experienced, and consequently, the Al industry is a notable consumer of refractory materials.

There are many physical and chemical parameters that strongly affect the corrosion resistance of refractories and these include grain size, the composition of the refractory and molten metal, the refractory density and its porosity [23]. In order to tolerate high temperatures, refractories should be thermally resistant when in contact with hot molten metals, slags, and fluxes [24]. Refractory materials, which normally include a high silica (SiO_2_) fraction, are used in molten Al furnaces because of their low thermal expansion coefficient, and the presence of SiO_2_ at high temperatures leads to low expansion variations. Where cracks exist in a refractory, fused silica is used to fill in the cracks at high temperature. Therefore, after cooling, the molten silica fills the cracks without changing the refractory volume, the density of the material increases, and the cracks are closed. Although the corrosion resistance of SiO_2_ is lower than that of Al_2_O_3_ when in contact with molten Al alloy, it is widely used in the refractory areas because of its low thermal expansion coefficient [25]. Higher Al_2_O_3_ to SiO_2_ ratio in the refractory composition enhances the corrosion resistance against Al attacks. Furthermore, some non-wetting additives, such as BaSO_4_, CaF_2_, MgF_2_, AlF_3_ are used to improve the corrosion resistance of the refractories in contact with molten Al alloys [26].

Therefore, the purpose of this review paper is to summarize and discuss progress reports available in literature, supplemented by some recent experimental results by our research group using different non-wetting additives to improve the performance of a mullite-based refractory. The modified materials were evaluated for their effect on the corrosion resistance of white fused mullite (WFM) materials in direct contact with a molten Al-Mg alloy. WFM was selected for this project because it has a high melting point (over 1800 °C), it exhibits excellent thermal stability, and it has low reversible thermal expansion and excellent thermal shock resistance when exposed to high temperatures. In addition, it displays a high corrosion resistance to many chemicals. Although there is significant diffusion of gases and volatile species from the molten alloy infiltrating the refractory lining through existing porosities, this paper only focusses on the penetration effects of the molten Al and Al-Mg.

Refractory materials are used in all steps of production of Al and its alloys, from the alumina calciners to the cast house furnaces and other metal handling equipment [22]. The Al industry is one of the biggest consumers of refractories in Canada, with Quebec province in particular being one of the preeminent producers of Al in the world. Therefore, Pyrotek Inc. (Canada), which is also based in Quebec and is a major producer of refractories for the Al industry, sponsored this study. Notwithstanding Al-Li alloys with corresponding corrosive chemical reactions, the attention of this paper is given to the corrosion of furnace refractories used in producing Al and Al-Mg alloys.

## 2. Application of Refractories in the Aluminum Industry

### 2.1. Refractory Selection Criteria

Since refractory materials have high melting points, fusing them is difficult except at extremely high temperatures. These materials are thermally stable and do not decompose easily when heated, and thus, they retain their chemical and physical properties at high temperatures [27]. Their melting point is the most critical physical property because it determines the temperature limit at which the material can be used without melting or decomposing [28]. Other important criteria used for selecting suitable refractory materials include density and porosity, mechanical resistance, thermal conductivity, thermal shock resistance and chemical resistance [11].

**Figure 1:**
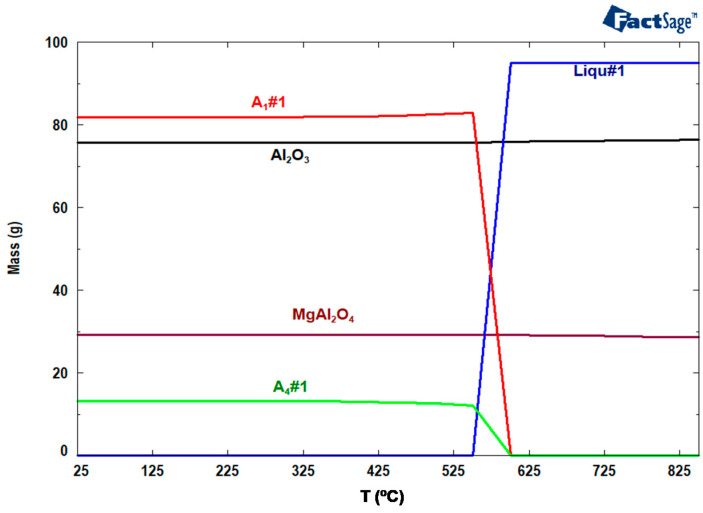
Equilibrium calculation of 3Al_2_O_3_·2SiO_2_ and molten Al-Mg alloy. Liq#1: liquid solution (81.79% Al + 13% Si + 0.11% Mg), A_1_#1: solid solution (81.8% Al + 1.3 × 10^−3^% Mg + 1% Si), A_4_#1: Solid solution (13% Si + 2 × 10^−5^% Al).

Some authors have observed that refractories in contact with molten Al alloys easily get corroded and degraded because of the extremely reducible behavior of the molten Al alloys [17]. Therefore, selection of the refractory material is a critical matter, which depends on the interaction between molten Al alloys and the refractory materials, as well as the nature of their reactions at high temperatures. There are some ceramic refractory materials that have excellent chemical and thermal stability when in contact with molten Al, and they include nitride-based materials, such as aluminum nitride (AlN) [29], silicon nitride (Si_3_N_4_) [30], and boron nitride (BN) [31]. Other examples involving yttria-based materials are Y_2_Ti_2_O_7_ [32,33] and nano scale coatings of Y_2_O_3_, which show high density, uniform structure and high hardness [34]. In fact, among all refractories used in the Al industry, Al_2_O_3_·SiO_2_ refractories are popular due to cost advantages, their ease of availability, and remarkable versatility, although they are prone to corrosion by molten Al alloys, especially Al-Mg. Knowledge of reaction thermodynamics is a prerequisite in the selection of suitable refractory materials for molten Al-Mg alloys, because it provides real insight into the reactivity potentials of the materials. Figure 1 is an image derived from FactSage^TM^ thermodynamic software, (Montreal, QC, Canada) [35] which is a theoretical calculation between 100 g of mullite (3Al_2_O_3_·2SiO_2_) when it comes into direct contact with 100 g of molten 5-wt.%Mg/Al between 850 °C and ambient temperature. The model confirms the potential formation of Al_2_O_3_ and MgAl_2_O_4_ that exists below 850 °C, accompanied by the reduction of SiO_2_ to release Si. It shows that about 13% of SiO_2_ diffuses from mullite to the molten Al alloy changing the liquid concentration from Al-(5-wt.%)Mg to 81.8% Al + 13% Si + 0.1% Mg. It also shows that 4.9% Mg out of the initial 5% reacts with mullite to form the spinel. By cooling, the liquid solution solidifies to A_4_#1 (13% Si + 2 × 10^−5^% Al) and A_1_#1 (81.8% Al + 1.3 × 10^−3^% Mg + 1% Si), indicating a total destruction from the corrosion of mullite. Therefore, choice refractory materials that should be used with molten Al alloy furnaces must have the following characteristics:

- They should have a low solubility in molten Al alloys;
- They should have excellent volume stability;
- They must be resistant to abrasion, oxidation, and reduction [23]; and
- They must lower the chemical potential difference between the refractory and the molten alloy [36].

### 2.2. Properties of Aluminosilicate Refractories

Typical refractories used in the Al industry are based on aluminosilicate refractories. However, their main limitation is corrosion as a result of the infiltration and reaction of the molten Al into the refractory. Consequently, the non-wetting properties of these refractory materials should be boosted against molten Al alloy to avoid infiltration. Applications of aluminosilicate refractories include insulations behind hot-face materials, furnace linings, and laboratory crucibles. These refractories are extensively used in melting and holding Al alloy furnaces due to their ease of availability and cost advantages [23]. Not only should refractories be resistant to high temperatures and thermal shocks [37], but should also be resistant to corrosion when in contact with the molten alloys and slag [38].

Some investigations into the corrosion kinetics of aluminosilicate materials under static and dynamic conditions have been conducted using molten 5-wt.%Mg/Al and the influence of SiO_2_ amount [39]. The researchers reported that the corrosion depth has a direct relationship with the SiO_2_ content in the aluminosilicate refractory during immersion tests in 5-wt.%Mg/Al at 850 °C. Therefore, the higher the SiO_2_ content, the greater the corrosion depth. Increasing the amount of SiO_2_ decreases the incubation time, which is defined as the time that a trace of corrosion (less than 1 mm) can be observed on a refractory by the unaided eye [39]. However, some authors observed that a content higher than 20-wt.% SiO_2_ did not show any remarkable difference in the incubation period [24]. The authors advanced the idea that perhaps the diffusion process that controls the refractory corrosion under dynamic conditions is more severe than under static conditions. Mullite (the only stable binary crystalline phase in the aluminosilicate phase diagram) belongs to the category of aluminosilicate refractories with compositions of different Al_2_O_3_ to SiO_2_ ratios ranging from 3Al_2_O_3_·2SiO_2_ to 3Al_2_O_3_·SiO_2_, and has an orthorhombic crystal structure consisting of oxygen vacancies [24]. When the SiO_2_ in the aluminosilicate refractories come into contact with molten Al, they form corundum, and therefore, the higher the Al_2_O_3_, the greater the corrosion-resistance to the molten Al alloy [40].

## 3. Corrosion of Refractories in Al Industry

### 3.1. The Origin of Corrosion

Different parts of the refractory linings experience different contact times with molten Al alloys, and consequently, they are exposed to different destructive mechanisms, such as thermal shock, mechanical impact, corrosion, and erosion. Among them, corrosion and erosion are the worst damages experienced, because the two have a severe effect on the purity of the resulting alloys and also on the lifetime of the refractory linings [41]. Therefore, one of the chief limitations in the production of Al alloys is the destructive behavior of Al and Mg in contact with refractories. There are two main failure mechanisms when molten Al alloys come into contact with refractories:

(i) Chemical corrosion, which is related to the penetration of molten alloys and reactions that lead to the dissolution of the refractory materials to form a new interphase layer [23], where the two main channels for the initial penetration of molten metal into the refractories are open pores and microcracks [24];
(ii) Erosion, which refers to a swift flow of molten alloy in the furnace, and if it includes some hard particles, the problem becomes worse, and mechanical wear occurs on the surface of the refractories [23].

These problems are tackled by making dense refractories that lack any form of porosity and dissolvable components in molten Al alloy, in order to improve the refractory life span [23]. Wear originating from corrosion as a result of refractory contact and reaction with molten metal alloy is accelerated by high temperature [42]. This is in addition to material losses and the synergetic effects between increased surface roughness, wear and corrosion processes [43]. In this process, dissolution, penetration, and reaction mechanisms occur between the liquid and solid phases [44]. These phenomena result in considerable changes in the microstructure, morphology, and composition of the refractory, and consequently altering the chemical and physical properties of the materials [45].

When two different metals contact each other at high temperatures, the formation of a new phase between them strongly depends on three factors: (i) their chemical potentials, (ii) the nucleation conditions at the start of the diffusion process, and (iii) the mobility of the elements in the refractory materials [45]. Therefore, a method to retard the corrosion is required. For example, in Fe-based crucibles, the addition of Al in the refractory components could diminish the discrepancy in their chemical potential, thereby decreasing the driving force towards corrosion [46]. Moreover, grain boundaries are normally regarded as the main diffusion paths in the corrosion of refractories, and the grain size is an essential parameter for the improvement of the corrosion-wear resistance of refractories [36]. Based on the amount of Mg in the Al-Mg alloy, MgO, MgAl_2_O_4_, or both (MgO, MgAl_2_O_4_) can be the product of the reaction between the Al alloys and the refractory, besides the presence of enough oxygen aiding the oxidation of the Al.

**Figure 2:**
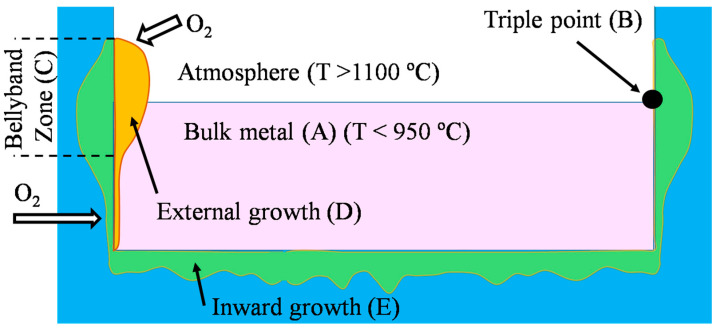
A schematic of internal and external corundum growth in Al-treatment furnaces modified after [47].

It is important to note that refractories in the Al industry should be resistant to the detrimental effects of the process, both physical and chemical. Physical impacts include mechanical abuse arising from scrap charging, thermal and mechanical shocks during skimming, cleaning and fluxing. The chemical effects culminate into wear, molten alloy attacks, liquid penetrations and corrosion, especially in the bellyband area (the triple point between molten liquid alloy, the solid refractory, and the gaseous atmosphere), as exemplified in Figure 2.

Zone A represents molten Al and the triple point B is where the liquid Al meets the solid refractory and air. The belly band zone C represents the region where the fluctuating meniscus of the molten metal meets the refractory. When molten Al reacts with atmospheric oxygen in belly band area, it builds external corundum at the interface between the atmosphere, aluminum bath, and the refractory, which is shown by zone D. With the penetration of molten Al into the refractory and its reaction with refractory oxides such as SiO_2_, it produces internal corundum represented by zone E.

There are two major undesirable products arising from the reaction between Al and the free silica of aluminosilicate refractory materials; that is, the spinel, and corundum, which cause spalling of the refractory walls and reduces their thickness [25]. This in turn affects the durability of the refractory materials and impacts negatively on the lifetime of the refractory linings, as well as the quality of the final Al product as a consequence of contamination from the impurities that migrate into the Al alloys [23]. Normally, corrosion is initiated by the molten Al alloy wetting the refractory surface, followed by the infiltration of the molten metal and subsequently the formation of a new phase at the interface of the refractory and the molten metals [24].

### 3.2. Corrosion Mechanisms

Since refractory linings are subjected to various degradation phenomena, such as thermal shock, mechanical impact, abrasion, corrosion, and erosion, corrosion is the worst case and impacts severely on both metal quality and refractory lifetime [48]. In short, it has been observed that aluminosilicate refractories in contact with molten Al alloy face mainly three problems: (a) penetration of molten alloy to the refractory, (b) side-wall build-up, and (c) silicon pickup by the metal [49]. The diffusion of Al and Si is a crucial factor in governing penetration rates.

When molten Al penetrates into the refractory, it reacts with SiO_2_ to produce corundum and free Si, as demonstrated in Figure 1 by FactSage^TM^, leading to a volume expansion, which exacerbates cracking. Furthermore, the ultimate lifespan of the refractories could be predicted by calculating the amount and rate of liquid penetration using models that incorporate both thermodynamic and kinetic studies [22]. From Figure 2, four main zones of corrosion in furnaces emerge:

(i) Zone A below the metal line that is continuously in direct contact with the molten alloy [50];
(ii) Zone C and D below and above the metal line, which is alternately exposed to the Al alloy and the furnace atmosphere [49];
(iii) The zone above the metal line, which is exposed to the furnace atmosphere and gases [49];
(iv) The triple point B between the refractory, the molten alloy and the air interfaces [51].

It has been reported that corundum is formed at the surface of molten Al alloys, due to the presence of the oxygen from the atmosphere, and that molten metal can flow through channels of the corundum [52]. When the corundum grows upwards, the molten alloy can reach the refractory wall above the metal line. A combination of high temperature and capillary action of the corundum growth provides an ideal opportunity for further penetration of metal alloys inside the refractory. Similarly, the molten alloy can diffuse through the open porosity of the refractory, with oxidation occurring in the pores [53].

#### 3.2.1. The Effect of the Porosity in the Refractory

It has been observed that the purity of any Al alloy under synthesis has a direct relationship with the characteristics of the refractory materials, such as their chemical and mineralogical composition, types of binders utilized [54], and their permeability [40]. If, for example, the permeability of the refractory to air increases, it will promote corrosion, since it allows gases such as oxygen and water vapors to diffuse through the refractory pores [55]. When molten Al alloy finally penetrates the refractory, it reacts with aluminosilicate to form corundum, and through a redox reaction, free Si is released [23]. The formation of corundum starts just below the bellyband and extends upwards, penetrating and sticking strongly to the porous refractories, and this makes it difficult to remove, and cleaning the furnace becomes a challenging and expensive process [56].

#### 3.2.2. The Effect of Corundum Formation

The two critical destructive mechanisms, which reduce the lifespan of refractories, are chemical attack (corundum growth or corrosion from flux addition) and mechanical damage (from ingot loading, cleaning practices, or thermal shock). Although the creation of the corundum layer in the refractory prevents further infiltration of the molten Al, the formation of corundum leads to an expansion in the refractory volume and, consequently, distortion and finally creating cracks [57]. There are two forms of corundum, internal and external:

(i) Internal corundum exists where the molten alloy penetrates into the refractory and reacts with the refractory oxides such as SiO_2_, corroding the refractory, while at the same time, corundum precipitates below the liquid metal line on the refractory surfaces, as illustrated in Figure 2. The reactions can be described through Equations (1) and (2) [58].
(ii) External corundum at the bellyband, which induces maximum corrosion to form corundum. Alloy penetration into the refractories is initiated by capillary action, and in the presence of atmospheric oxygen produces corundum, which adheres severely to the refractory’s surfaces [40]. With the presence of Mg in molten Al, the corrosion process accelerates and reduces the refractory oxides more aggressively than with Al alone.

#### 3.2.3. The Effect of Molten Alloy Infiltration

Thermodynamically, the reaction between SiO_2_ and Al is possible at all temperatures above the melting point of Al. At the surface, due to the direct contact between molten Al alloy and the refractory, the corrosion process begins with wetting of the refractory. This is then followed by a reaction that creates an interface with a different chemical composition. In this process, the molten Al diffuses into the refractory through existing cracks and open pores. The amount and rate of diffusion is related to the pore size, the temperature, composition, and texture of the refractory materials, as well as the type of alloy under synthesis [59].

#### 3.2.4. Effect of Enhanced Wetting on Refractories

One of the most critical interfacial phenomena occurring at the refractory surface is the wetting process, because it facilitates the penetration of the molten alloys through the open pores and then initiates various chemical reactions within the refractory [49]. Results from various studies show that the principal genesis of corrosion is related to the wetting, penetration, and reaction of Al alloys with the refractory. It has been observed that the penetration of the molten Al alloys is highest at the bellyband. For instance, at the onset of corrosion in aluminosilicate refractories, the SiO_2_ is reduced by molten Al to Si, which is accompanied by a negative volume change [59], by as much as 26% volume reduction [60]. Therefore, this volume contraction may generate cracks that allow further metal penetration into the refractory [39], and the amount of Al alloy diffusing into the unreacted refractory controls the rate of corrosion [49]. Furthermore, an increase in air permeability of the refractory will raise the probability of corrosion occurring by molten alloys, and allow gases such as oxygen and water vapor to diffuse through the refractory pores [55].

### 3.3. Corrosion of Typical Refractories in Al Industry

#### Aluminosilicate Refractories and Corrosion

In aluminosilicate refractories, the molten Al alloy on the surface reacts with the atmospheric oxygen in air to form a porous corundum film, which has channels that direct the molten alloy to the surface of the refractory, a process that is intensified at the bellyband [59]. When the metal penetrates the refractory to reduce SiO_2_, this part of molten Al is gradually saturated by free Si. However, the SiO_2_ does not release Si into the metal bath spontaneously, because there is a Si concentration gradient created, and the diffusion rate of Si to the metal bath controls the corrosion kinetics [39]. The reaction between aluminosilicate refractories and molten Al is almost immediate, and it is possible to prove it by measuring the amount of Si in the molten alloy, with the results showing that the amount of Si increases significantly during the first 2–3 days [61].

The corrosion of aluminosilicate refractories initiates the decomposition of 3Al_2_O_3_·2SiO_2_ to Al_2_O_3_ and SiO_2_. Then, the SiO_2_ is reduced by Al metal to form the primary corundum (α-Al_2_O_3_) and releases the free Si to molten Al [60]. When the molten Al-Mg alloy comes into contact with the refractory, MgO, MgAl_2_O_4_ (spinel), and MgSiO_3_ are formed. The formation of spinel causes a 17% volume expansion, which is accompanied by spalling of refractories, and by creating cracks, a higher penetration of molten alloy is achieved [22]. During the process, the formation of additional metastable phases like ɳ- and ɵ-Al_2_O_3_ or suboxides like AlO and SiO are observed [62].

The primary Al_2_O_3_, which is obtained from the decomposition of 3Al_2_O_3_·2SiO_2_, builds a scaffold for the precipitation of spinel and secondary corundum. The Al_2_O_3_ that is formed from the reduction of SiO_2_, at first is in a metastable phase (ɳ, ɵ-Al_2_O_3_), but later transforms to the thermodynamically more stable phase (α-Al_2_O_3_) [62]. Since there is a competition towards the formation of MgO, MgAl_2_O_4_ and Al_2_O_3_ because SiO_2_ has a higher affinity for Mg in comparison to Al, the reaction products are in direct relation to the Mg concentration in the Al-Mg alloy. Replacement of SiO_2_ by MgO, MgAl_2_O_4_ or Al_2_O_3_ increases volume contractions by about 18%, 27%, or 38%, respectively [63]. Various reactions that occur in the above-mentioned process are summarized in Table 1, with values recorded to one decimal point.

Based on the tabulated information, Equation (1) indicates the oxidation reaction of Al metal to form alumina, while Al can reduce SiO_2_ to produce corundum according to Equation (2). Mg can reduce alumina to form the MgAl_2_O_4_ spinel based on Equation (3), or it can also reduce SiO_2_ to form MgO and free Si as given in Equation (4). Free Si may also be produced by the reaction between the molten Al and the aluminosilicate refractory, according to Equation (5), while Equation (6) shows the oxidation reaction of Mg in air to form MgO. Equation (7) indicates that both metallic Al and Mg can combine with SiO_2_ to form the MgAl_2_O_4_ spinel, and based on the Gibbs free energy provided in Equation (8), it is comparatively less likely for the spinel to form by the reaction between MgO and Al_2_O_3_. Furthermore, from Gibbs free energy values, it is easier to reduce the SiO_2_ in a refractory (Equation (9)), than the Al_2_O_3_ (Equation (10)), and both produce MgO and free Si or Al, respectively.

From Equation (11) of positive Gibbs free energy, it is not possible for molten Al to reduce MgO. However, the presence of atmospheric oxygen can enhance the spinel formation based on Equation (12). In the absence of air, the probability of excess Mg reacting to decompose the spinel into MgO and forming free Al is very remote, according to the small Gibbs free energy value given in Equation (13). From Equation (14), there exists some possibility that the presence of Mg in the molten alloy can react with the free Si released from Equation (2) to produce MgSi_2_, although the probability is low. On the other hand, Equation (15) shows that celsian (BaAl_2_Si_2_O_8_), which is a good anti-corrosion material in the refractories, can be decomposed to produce corundum and free Si. The same is possible for mullite (Al_6_Si_2_O_13_), according to Equation (16). From the small ∆G^o^ values, Equations (17) and (18) indicate the formation of anorthite as a stable phase, since it can hardly decompose to produce corundum.

From the above equations, the FactSage^TM^ modelling provided in Figure 1 correctly predicts the reaction between mullite (3Al_2_O_3_·2SiO_2_) and molten 5-wt.%Mg/Al. Equation (2) shows that the SiO_2_ in mullite produces corundum. With negative ∆G^o^ (as −528 kJ), it means that the reaction will definitely occur. Based on the model, Al_2_O_3_ (corundum) easily forms at all temperatures (black line). Similarly, from Equation (5), corundum forms when mullite reacts with molten Al (∆G^o^ = −1033 kJ). In the presence of Mg, spinel forms at all temperatures (maroon line). In Equation (3), when Mg reacts with Al_2_O_3_ arising from Equation (2), the ∆G^o^ is −208 kJ; while in Equation (7), the concurrent presence of Al and Mg reacts with the SiO_2_ in mullite to form the spinel (∆G^o^ is −422 kJ). The negative Gibbs free energy values mean that the reactions will easily take place. This implies that the model sufficiently predicts the destruction of pristine mullite when in contact with molten Al-Mg alloy.

There are two practical ways of avoiding the corrosion of refractory materials that are in direct contact with molten Al alloys:

(i) Chemical means, by the addition of non-wetting additives (NWA) as components of the refractory materials [71]; and
(ii) Physical means, through surface modification and densification by, for example, creating a protective coating on the surface of the refractory, which comes in direct contact with the molten alloy [40].

Since wetting plays a crucial role in initiating the corrosion process, followed by infiltration of the molten metal into the refractory, and then a reaction between molten alloy and the refractory, in this work, an approach is developed where NWAs are added to refractories that come in direct contact with the molten Al alloys. The central substrate under review in this work is based on mullite, and the anti-wetting additives are incorporated in order to improve the corrosion resistance of the refractories by forming new phases.

Refractories produced with mullite have attracted much attention, especially for high temperature applications; not only in the Al industry, but also among others, the steel industry, because of their high thermal stability and excellent resistance to thermal shock, creep, and corrosion. Mullite can be added to the initial refractory paste or generated in situ during firing, which intrinsically creates different microstructure from that formed by mullite when added initially [37]. It has been observed that the presence of mullite minimizes the corrosion of alumina refractories [71].

### 3.4. Wettability and Surface Tension

#### 3.4.1. Origin of Wettability Theory: Young’s Regime

Thomas Young introduced the relationship between the contact angle and surface energy of different interfaces using Equation (19) [72], and this definition has been used to quantify the wetting behavior of surfaces.

(19) $$\cos\theta_{Y}=\frac{\left(\gamma_{SG}-\gamma_{SL}\right)}{\gamma_{LG}}$$

where:

*θ_γ_* = Young’s contact angle at equilibrium, and

*γ* = Surface energy for liquid-gas (LG), solid-gas (SG), and solid-liquid (SL) interfaces

**Figure 3:**
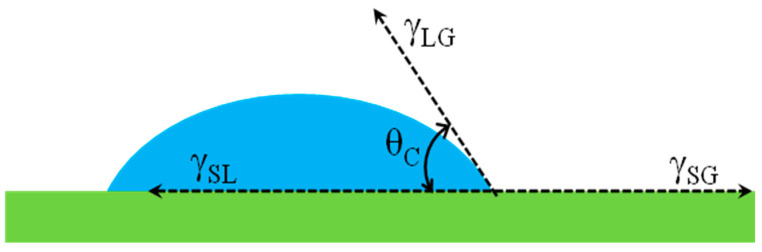
Contact angle as defined by Young’s equation; figure modified after [72].

Therefore, the wettability of a surface by a liquid is measured based on the contact angle between the solid and the liquid droplet, as shown in Figure 3. According to Young’s equation in our situation, it was expected that the enhanced wetting of refractories would occur when the contact angle between molten Al alloy and the refractory substrate was lower than 90°. If the angle was greater than 90°, then the refractory became non-wetting when in contact with a droplet of molten alloy.

Since the wettability of a surface is governed by the chemical properties and the microstructure of the surface, which is mainly determined by its free surface energy or surface tension (γ_SG_), the greater the free surface energy, the easier it is for the liquid to spread upon the surface and vice versa [73]. Equation (19) is therefore valid for perfectly flat and uniform surfaces, which limits its application for real surfaces. Besides, other external forces such as change of properties with increasing temperature can enhance wetting [74].

#### 3.4.2. Wetting Heterogeneous Surfaces: The Wenzel Regime

Since Young’s equation is applicable for ideal surfaces that are free from inhomogeneities, the study of wetting behavior puts into consideration the surface roughness of refractories. Wenzel and Cassie–Baxter models account for surface defects in the wetting of rough and heterogeneous materials [73]. In the Wenzel state, which Robert Wenzel first introduced in 1936 [75], the relationship between the contact angle and surface roughness is highlighted. It is assumed that the liquid completely penetrates into the surface roughness grooves, for which reason this surface is considered as a wetted regime [76]. It is further asserted that surface roughness increases the contact angle when it is higher than 90°, which implies that hydrophobicity is enhanced by an increase in surface roughness [77].

#### 3.4.3. Penetration of a Liquid on a Rough Surface: Cassie–Baxter Regime

About eight years after the postulation of the Wenzel theory, Cassie and Baxter [78] both investigated the contact angle of a liquid drop in contact with a rough surface before the liquid penetrated into the surface grooves, and they predicted the relationship between roughness and contact angle. The Cassie–Baxter wetting regime is a quasi-stable state, which, over time, transforms to the Wenzel state [79]. In reality, a combination of these two regimes is usually observed for example, as a water drop partially diffuses into the grooves of a solid surface [80,81].

#### 3.4.4. Surface Wetting by a Moving Droplet: Hysteresis Contact Angle

**Figure 4:**
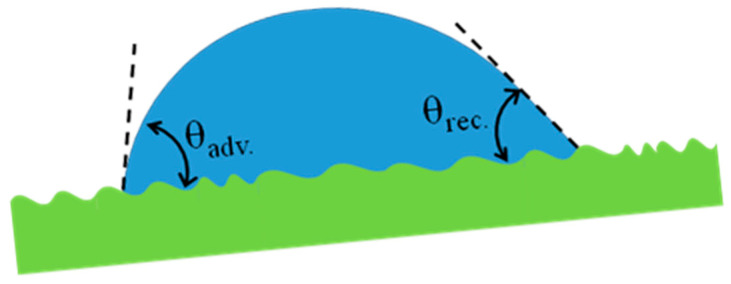
Dynamic contact angles at the interfaces of a moving droplet modified after [71].

On most occasions, contact angles will be measured and derived for a static water drop, which is not in motion. However, for a moving water drop, it can display dynamic contact angles, with some differences in the advancing (front side) and the receding (rear side), which defines the hysteresis contact angle (HCA), as shown in Figure 4 [72]. HCA is one of the critical factors in the roll-off behavior of a liquid. Lower contact-angle hysteresis causes a lower adhesion force between the droplet and the surface, and results in easier sliding of the droplet on the surface [73]. The magnitude of HCA is affected by different factors, such as surface roughness, surface chemical heterogeneity, droplet size effect, surface deformation, and adsorption/desorption phenomena [76]. Among them, the HCA is more affected by surface roughness, where it changes a homogeneous surface to a heterogeneous one [82]. It has been observed that the roughness parameter enhances the hysteresis and static contact angle of hydrophobic surfaces. However, in some cases, an increase in the static contact angle lowers the HCA. This decrease in HCA can qualitatively be explained by the switch from Wenzel to Cassie–Baxter regime, with air trapped at the macroscopic liquid-solid interface [83].

#### 3.4.5. Wettability and the Triple Line

Another factor whose influence has a vital role in the wetting phenomenon is the triple line, which is defined as the line where the solid, liquid and air phases meet [74]. It has been illustrated that there is a strong relationship between the HCA value on very rough surfaces and triple line characteristics such as shape and continuity [84]. As a case study, three hypothetical roughness topologies were considered, and the best case for minimizing the HCA was obtained from the use of thin pillars creating a discontinuous triple line [85]. It was observed that surface roughness generated by slender pillars with the smallest thickness in proportion to the height and periodic spacing displayed the best hydrophobic properties. Decreasing the ratio between the thickness of the pillar and its height results in a higher-energy barrier system, which means that the roughness height has a significant impact on the magnitude of the energy barrier as it is at maximum, for the microstructure with very tall and slender pillars. So, nanopillars with suitable spacing render great superhydrophobic surfaces. It can therefore be concluded that the wettability of a surface depends on its physico-chemical properties in addition to its micro or nano roughness. These two factors determine the extent of adhesive forces between a liquid droplet and the surface [86].

#### 3.4.6. Surface Wettability and Interface Formation

In the metal industry, wetting by molten alloy must be controlled to protect refractories against corrosion, which is impacted by factors, such as impurities, the alloying elements in the melt and the surface roughness of the refractories [87]. Since, in many materials processing techniques such as casting, the molten metals and refractories are in direct contact, the characteristics of the final product are profoundly affected by the high-temperature properties of molten metal, and the interfacial wetting/reaction phenomena. Therefore, the interaction between the molten metal and the substrate is one of the critical factors considered when choosing suitable materials and processing parameters [88]. For the Al-SiO_2_ system, there is a significant reduction in the droplet volume during the reactive wetting, and this is due to the formation of a new interfacial compound between Al and SiO_2_ to produce Al_2_O_3_-Al(Si) composite. Modeling has shown that the final contact angle is governed by the interfacial reaction, and that the wetting process by contact angle only is not sufficient [87].

Some researchers who investigated the reactivity of molten Al with some ceramic oxides such as Al_2_O_3_, SiO_2_ and mullite, have demonstrated that a strong relationship between wettability and reactivity in the system cannot be established [89]. However, other researchers have reported that the wetting of a ceramic substrate is usually accompanied by an interfacial reaction between the metal and the ceramic. Hence, a new composition was formed during the reaction at the interface, with a strong effect on the magnitude of the interfacial free energy, and subsequently changes in the contact angle [90]. It has been observed that in the reactive wetting of molten Al on different α-Al_2_O_3_ surfaces, the process is reaction-limited and the spreading rate is dominated by the change in the solid–liquid interfacial free energy per unit time [91].

#### 3.4.7. Improvement of Aluminosilicate Corrosion Resistance by Non-Wetting Additives

Today, researchers are focusing on a new generation of refractories, in order to decrease the infiltration and attack from molten Al alloys. To achieve this goal, the use of some NWAs has been attempted, which includes aluminum phosphate (AlPO_4_) [92], vanadium pentoxide (V_2_O_5_) [93], barium sulphate (BaSO_4_) [94], strontium sulphate (SrSO_4_) [85], calcium fluoride (CaF_2_) [95], aluminum fluoride (AlF_3_), aluminum titanite (Al_2_TiO_5_) [95], calcium silicate (wollastonite) and boron nitride (BN) [96]. The NWAs lessen the wettability capacity of refractories in contact with molten Al alloy, and after reaction with SiO_2_, they produce a more stable phase than free Si, which improves the corrosion resistance of the materials. Some additives containing fluorides such as AlF_3_ and CaF_2_ also act as mineralizers, and during the high-temperature calcination of aluminosilicate refractories, they favor the formation of mullite [96].

Furthermore, when molten Mg is in contact with AlF_3_ or CaF_2_, it forms MgF_2_, which can also act as a non-wetting agent [97]. The presence of wollastonite in refractories improves their corrosion resistance by reducing the permeability of the materials [98]. Incorporating BN improves the corrosion resistance of the refractories by the formation of the thermally stable aluminoborate phase, and it also lowers the solubility of boron in the molten Al, which drastically increases the corrosion resistance of andalusite refractories when in contact with molten Al alloys [99].

For BaSO_4_ additives, it has been reported that BaSi_2_Al_2_O_8_ forms, and therefore, the amount of free Si in the matrix decreases to improve the corrosion resistance of the refractory [70], making BaAl_2_Si_2_O_8_ exhibit good corrosion resistance when in contact with molten Al alloy [100]. The most effective application of BaSO_4_ in protecting the refractory matrix against corrosion is when particles in the size range of less than 50 Tyler mesh are used [55]. The reaction of barite (BaSO_4_) with Al_2_O_3_ and SiO_2_ in aluminosilicate refractories forms a stable and less reducible phase of celsian (BaAl_2_Si_2_O_8_) and/or hexacelsian, during the firing process, which creates a barrier against increased penetration of molten Al alloy [65]. It should be considered that BaSO_4_ is the most effective NWA in the firing temperature between 815 °C and 1050 °C, but it has been shown that, as the temperature rises to more than 1050 °C, the BaAl_2_Si_2_O_8_ phase becomes undetectable in the refractory, and it loses its efficiency [101]. This is attributed to either the phase transformation of celsian (BaAl_2_Si_2_O_8_), or its decomposition based on Equation (15) [102], when in contact with molten Al alloy.

For AlF_3_ and CaF_2_, this temperature decreases to less than 950 °C. Anorthite (CaAl_2_Si_2_O_8_), which is similar to BaAl_2_Si_2_O_8_, characterizes the CaO-ceramics and results in a dense structure and thus, the reaction in anorthite predominantly advances through solid diffusion. Most of the Ca is concentrated in the β-Al_2_CaSi_2_ phase formed, adhered at the interface [103].

## 4. Experimental Methods and Analysis

### 4.1. Experimental Procedure

To tackle the corrosion issues, researchers have proposed two solutions. The first one consists of the addition of non-wetting additives to the mixture of the refractory materials. These additives minimize corrosion by reducing the wettability of refractories in contact with the molten metal. The second solution involves coating the refractories with thin films to protect them from chemical and physical reactions. The reason why the industry can use coatings instead of modifying the substrate composition is to improve the surface functional performance, and to expand the lifetime of the refractory substrates by decreasing the wear due to abrasion, erosion and/or corrosion. An increase in profitability is expected as the coatings allow the use of low-cost based refractory materials [104].

In this work, mullite samples whose composition includes the presence of 3Al_2_O_3_·2SiO_2_ were investigated for their corrosion resistance behavior. An attempt to lower the wettability of the white fused mullite (WFM) refractory was made by the incorporation of NWAs, and their mechanical properties and corrosion resistance were evaluated and characterized. The objective of this research project was to find and develop new refractory materials that can be used in the Al industry for lengthening the life span of the refractories that come into direct contact with molten Al alloy.

The hypothesis advanced in this study involves evaluating the impact of adding NWAs to increase the corrosion resistance of the refractories. It is thought that the refractory materials get damaged due to the diffusion of the molten alloy through their pores, and subsequently react with the materials. Anti-wetting additives improve the corrosion resistance by the formation of new phases that create a barrier, which reduces the diffusion rate of molten Al alloy through the existing pores of the refractory materials. Since it is suspected that the formation of the MgAl_2_O_4_ spinel is the leading cause of cracking in the refractories, the NWAs are seen to inhibit the spinel formation and this therefore prevents the cracking process.

### 4.2. Materials and Methods

#### 4.2.1. Materials and Reagents

The proposed refractory family used in this work is the white fused mullite (WFM), whose composition and particle size are shown in Table 2, which also includes the company name of suppliers and other ingredients used.

Additives used to improve the non-wetting capacity of the WFM refractories included CaF_2_ from VWR-Anachemia, Montreal, QC, Canada (particle size: 44 µm), BaSO_4_ from EXbar, Houston, TX, USA (particle size: 44 µm), Wollastonite from NYCO Minerals, Willsboro, NY, USA (particle size: 37 µm), and Secar^®^71 (calcium aluminate) cement from Kerneos Inc., Chesapeake, VA, USA (particle size D50, 73.4 µm).

#### 4.2.2. Materials Synthesis

The WFM supplied by Pyrotek Inc. (Sherbrooke, QC, Canada) was modified by NWAs (2-wt.% CaF_2_, BaSO_4_, Wollastonite, Secar^®^71 cement, and a mixture of CaF_2_ and BaSO_4_). The microstructure components of the samples were mainly mullite and alumina. Secar^®^71 cement was added to one sample to facilitate the formation of anorthite. In the production of WFM, both large and fine particles were necessary in order to produce a dense refractory. Other studies have established the critical role of adding minute quantities of BaSO_4_ to high alumina content refractories, where 1-wt% of BaSO_4_ produces anorthite due to the reaction of the calcium cement phases with the refractory constituents [70]. Raising the BaSO_4_ content to 5-wt.% forms barium silicates instead of anorthite, and the addition of more than 10-wt.% BaSO_4_ creates celsian phase. In this work, four steps were involved in making the refractory bricks during the modification, and it required Ludox1144 as the liquid medium:

(i) Step 1: Mixing of precursors
  (a) Half of the total amount of Ludox 1144 was poured in the mixing pot;
  (b) Predetermined mass of the powder (WFM + 2-wt.% NWA) was added;
  (c) The rest of Ludox 1144 was added;
  (d) The mixture was stirred for 5 min;
  (e) The final mixture was then tested by determining the flow rate.
(ii) Step 2: Mold casting
  (a) The mixture was poured into a mold set on a vibrating table to level the concrete;
  (b) A piece of plastic was placed on top of the mold.
(iii) Step 3: Setting and curing
  (a) After 16 to 18 h, the cast was removed from the mold;
  (b) Samples were placed in a plastic container and covered with a damp cloth for 1 day;
  (c) The samples were further kept in open air for 1 day.
(iv) Step 4: Firing process;

The samples were calcined at various temperatures, applying the firing cycle in Table 3:

### 4.3. Materials Testing: Alcan Immersion Corrosion Test

This test is routinely used to evaluate the suitability of refractories for applications in melting and holding furnace linings. It is also used to determine the resistance of metal penetration in the furnace linings. In this study, the Alcan immersion test was used to evaluate the corrosion resistance of the samples under investigation performed at 850 °C for 96 h, to determine the extent of molten Al metal penetration [63]. For this purpose, two refractory samples of size 51 mm × 25 mm × 25 mm were placed in a clay-bonded graphite crucible including 2 kg of molten Al-(5-wt.%)Mg alloy, in a vertical electrical furnace. Since Mg is volatile, 40 g Mg was added to the molten alloy every day to keep its concentration constant. After the high temperature testing, the samples were taken from the cup and sectioned in order to evaluate (a) the level of cracking in the refractory, (b) the extent of metal infiltration into the refractory, and (c) the degree of metal adherence.

### 4.4. Materials Characterization

#### 4.4.1. Optical Microscopy

Fresh samples and those tested by the Alcan immersion test were analyzed for corrosion by an optical microscope (Keyence VHX-6000, Mississauga, ON, Canada).

#### 4.4.2. X-ray Diffraction (XRD) Analysis

X-ray diffraction (XRD) analysis is a valuable method in distinguishing different phases and crystallite sizes in powder and coated samples. The samples were analyzed before and after the corrosion test on a “Philips Panalytical X’pert PRO MRD” X-ray diffractometer (Almelo, The Netherlands), using Cu Kα1 radiation with a wavelength, λ = 1.54 Å, in the 2θ-angle range from 10–90°, at a scanning speed of 0.04° 2θ-angle per min and a step size of 0.02° and step time of 0.5 s.

#### 4.4.3. Scanning Electron Microscopy (SEM)

Microscopic imaging by SEM coupled with energy dispersive X-ray spectroscopy (EDX) were used to investigate the morphology, structure, particle size and composition of the materials. In this work, SEM imaging and analysis was done on a “Hitachi S-4700” Field-Emission Scanning Electron Microscope (Tokyo, Japan), equipped with an EDX X-Max Oxford spectrometer (Tokyo, Japan).

#### 4.4.4. Wettability Test

Wettability of the samples was studied using the Krüss Advance goniometer model DSA25E (Hamburg, Germany). In order to measure the surface energy of the samples at 25 °C, Van Oss theory was applied [105], because it works best for inorganic surfaces [106]. In the approach, diiodomethane (CH_2_I_2_) was used as the non-polar liquid, while water (H_2_O) and formamide (CH_3_NO) were used as the polar liquids [107]. In the Van-Oss-Goods theory represented by Equation (20), the surface energy calculation is dependent on the contact angle between the liquid and the solid materials, which are related by Equation (21) [108].

(20) $$\left(1+\cos\theta \right)\gamma_{L}=2\left(\sqrt{\gamma_{S}^{LW}\gamma_{L}^{LW}}\;+\sqrt{\gamma_{S}^{+}\gamma_{L}^{-}}+\;\sqrt{\gamma_{S}^{-}\gamma_{L}^{+}}\right)$$

(21) $$\gamma_{S}=\gamma_{S}^{LW}+\gamma_{S}^{AB}=\gamma_{S}^{LW}+2\sqrt{\gamma_{S}^{+}\gamma_{S}^{-}}$$

where *θ* is the contact angle;

*γ_L_* and *γ_S_* are the surface tensions of the liquid and the solid, respectively;

$\gamma_{L}^{LW}$ and $\gamma_{S}^{LW}$ are the apolar or Lifshitz–van der Waals (LW) interactions;

$\gamma_{S}^{AB}$, $\gamma_{L}^{+}\gamma_{S}^{-}$ and $\gamma_{S}^{+}\gamma_{L}^{-}$ are polar or Lewis acid–base (AB) interactions for liquid (L) and solid (S).

In this experiment, polycarbonate was used as a reference material in the analysis and the surface energy was found to be 45.5 mJ·m^−2^, which is in agreement with literature data [109]. Since surface energies of solids are usually measured at room temperature, for most adhesion energy works, they are assumed to be similar at elevated temperatures [110].

## 5. Results and Discussion

### 5.1. Materials Testing

**Figure 5:**
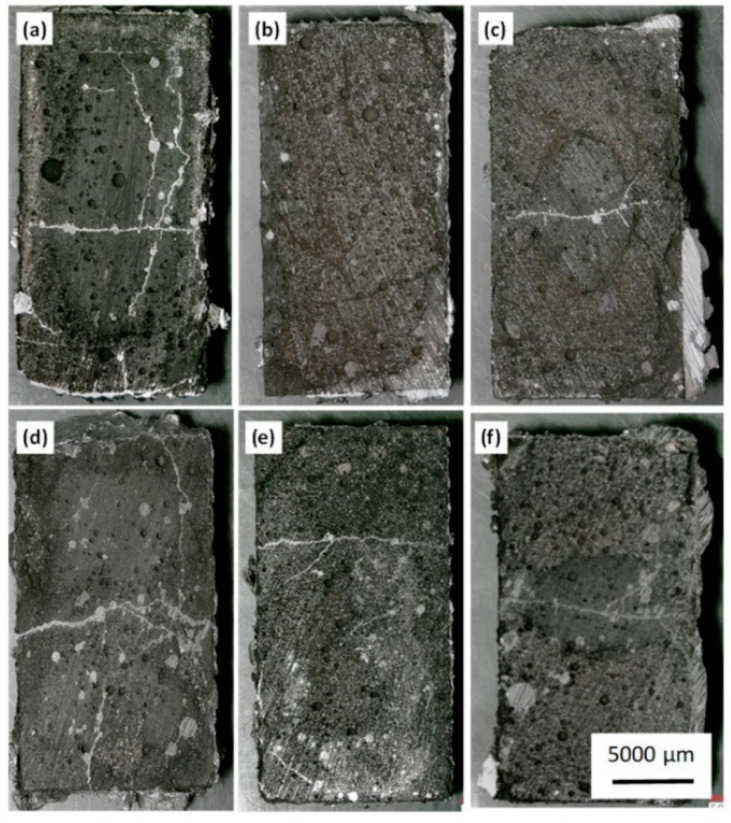
Optical microscopy images of samples after Alcan immersion test for (**a**) pristine WFM showing cracks and diffusion of Al; (**b**) addition of CaF_2_ and BaSO_4_ to WFM which prevents cracking; while the rest indicate cracking after addition of (**c**) Secar^®^71; (**d**) BaSO_4_; (**e**) CaF_2_ and (**f**) Wollastonite.

Figure 5 shows the optical microscopy image of the samples after the corrosion test. Two distinct corrosion zones were observed. The first zone arises from the direct reaction occurring at the interface between the sample and the molten metal, while the second zone is by infiltration of the molten Al-Mg alloy into the cracks formed in the samples.

The image of the plain WFM shown in Figure 5a reveals that the entire sample was totally corroded, with a reaction occurring at the interface and the molten Al-Mg penetrating the sample through the cracks. By adding NWAs, the corrosion resistance increased, and this can be observed from Figure 5b, where the addition of both CaF_2_ and BaSO_4_ to the pristine WFM limited the corroded area. The addition of Secar^®^71, BaSO_4_, CaF_2_ and Wollastonite to the pristine WFM did not improve the corrosion resistance of the WFM, as shown by the cracks in Figure 5c–f, respectively. Samples promoted using a mixture of both CaF_2_ and BaSO_4_ presented better corrosion resistance when compared to the samples with individual NWAs. No corrosion was observed for the entire sample, except for a small interfacial zone around the sample surface. Although there were some cracks in the sample, the Al alloy could not diffuse into the sample cracks.

### 5.2. Mechanical Tests

Table 4 indicates the mechanical characteristics of the modified samples after the addition of the NWAs. The test parameters in the three-point flexural and compressive strength analysis were selected according to ASTM C133-97. The samples’ sizes for flexural test were 51 mm × 51 mm × 228 mm and for the compressive strength test were 51 mm × 51 mm × 51 mm. Furthermore, the loading rate for the flexural strength test was 1.3 mm·min^−1^, and for compressive strength 13,608 kg·min^−1^. It was observed that the inclusions weakened the flexural strength of the pristine WFM sample, and the same applies to the Young’s modulus, where the additives introduced in the sample seemed to create interruptions in the continuity of the phases. Changes in the phase composition of the WFM + 1-wt.%BaSO_4_ + 1-wt.%CaF_2_ sample were perceived to inhibit corrosion by interrupting the propagation of cracks in the refractory. It was observed that samples containing CaF_2_ were more brittle and showed lower flexural strength.

### 5.3. Characterization

#### 5.3.1. XRD Results

**Figure 6:**
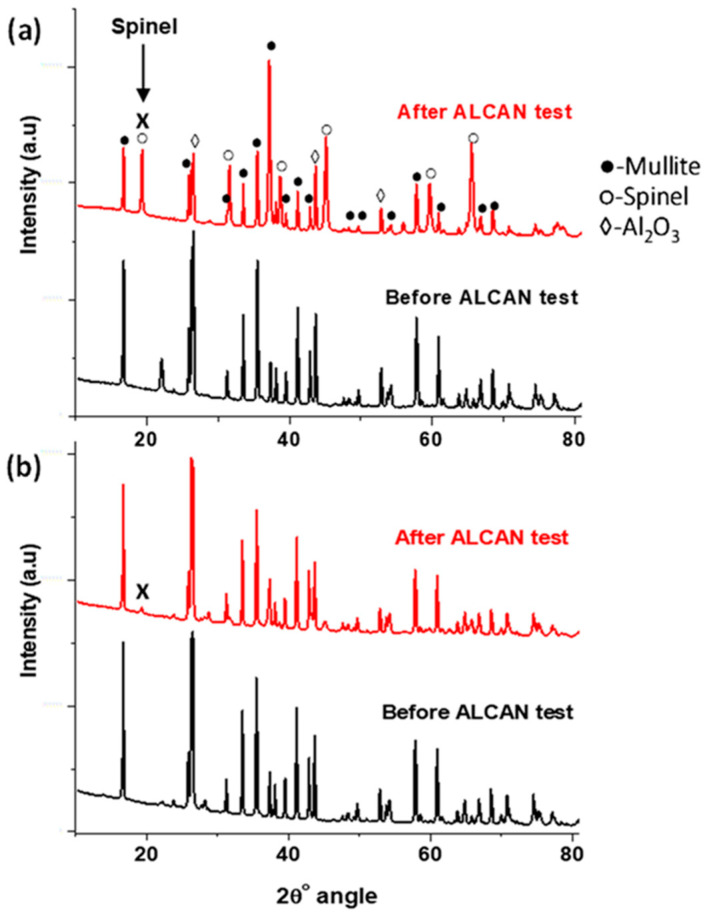
XRD patterns of (**a**) pristine WFM before and after Alcan immersion test showing formation of spinel (peak X); and (**b**) addition of CaF_2_ and BaSO_4_ to WFM prevents spinel formation.

The pristine WFM contained mullite, alumina and SiO_2_ phases, and after the corrosion test, it was found to contain an additional spinel (MgAl_2_O_4_) phase, as shown in Figure 6. The XRD peak present at 22° (2θ angle) in the pristine WFM, before the Alcan test shown in Figure 6a disappears after modification with NWAs, as seen in Figure 6b. This peak is related to the presence of crystalline SiO_2_ in the refractory. The crystalline SiO_2_ reacts with the additives to form more corrosion resistant phases, such as anorthite [66] or barium aluminosilicate [59]. The ICDD cards used to identify the phases include: [98-000-2103] for mullite (3Al_2_O_3_·2SiO_2_), [98-001-3213] for SiO_2_, [98-000-0174] for corundum (Al_2_O_3_), and [98-000-4595] for spinel (MgAl_2_O_4_).

It is suspected that the formation of the spinel and corundum is the origin of the cracks in the samples as some researchers have explained [63]. The XRD analysis in Figure 6a shows that pristine WFM contained the spinel phase (identified with the peak labelled X), and it only appears after the Alcan immersion test. However, with the addition of both CaF_2_ and BaSO_4_, the formation of the spinel phase was significantly suppressed, as indicated in Figure 6b, which improved the corrosion resistance of the sample. 

**Figure 7:**
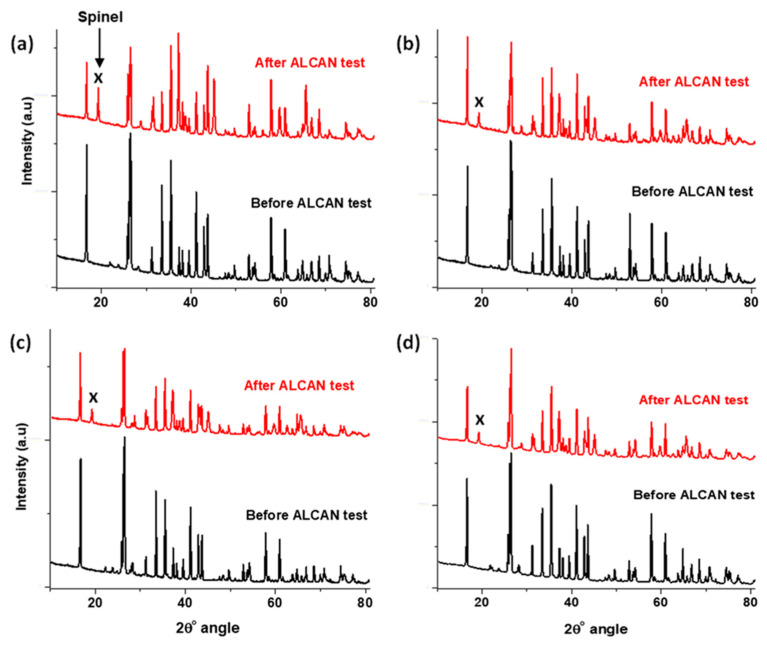
XRD patterns of pristine WFM with addition of (**a**) Secar^®^71; (**b**) BaSO_4_; (**c**) CaF_2_; and (**d**) Wollastonite before and after the Alcan immersion test, showing spinel formation (peak X).

In both Figure 6 and Figure 7, the XRD analysis indicates that all the samples contained the spinel phase, which led to the formation of the cracks, as seen in Figure 5. The addition of Secar^®^71 cement to WFM proved inconsequential. Comparatively, by observing the intensity of the peaks, this sample was the worst performer, because it contained the highest amount of the spinel phase. The other non-wetting additives, such as wollastonite, CaF_2_ and BaSO_4_ produced less quantities of the spinel phase.

Equations (1) and (2) show the circumstances under which Al_2_O_3_ may be produced, which in turn generates the spinel as shown in Equation (3). According to Equations (4) and (6), the presence of oxygen in the refractory as well as in air at the triple point reacts with Mg. This produces MgO that facilitates the formation of spinel, based on Equations (7) and (8). Since it is suspected that the production of the MgAl_2_O_4_ spinel accelerates the cracking and degradation of the refractories, any process that halts the formation of the spinel will be beneficial to the protection of the refractories. It was observed that the intensity of Al_2_O_3_ in the XRD patterns dropped after the Alcan immersion test, possibly because it was consumed in the production of MgAl_2_O_4_. The addition of NWAs was meant to inhibit the spinel formation, which was only successful with the combination of BaSO_4_ and CaF_2_. Table 5 provides a summary of the phases identified in the refractories after the Alcan test.

It is clear that none of the NWAs (including BaSO_4_ and CaF_2_ separately) obstructed the spinel production. However, where the two were used in combination, many phases such as anorthite (CaAl_2_Si_2_O_8_), MgF_2_ and CaS, which contain significant amounts of both Al and Mg elements were formed. These findings confirm certain studies, which indicated that samples containing 3% CaF_2_ and 2% BaSO_4_ displayed the best wetting resistance to the Al-alloy [70]. The concomitant presence of BaSO_4_ and CaF_2_ prevented the two elements from feeding into the formation of MgAl_2_O_4_ spinel, and in so doing, improved the corrosion resistance of the refractories. From the XRD data, the intensity of the spinel peak (X) in Figure 6 and Figure 7was extracted and compared, and they appeared in the respective ratio of 1:2:3:3:4:5 in the ascending order of: “WFM + BaSO_4_ + CaF_2_ < WFM + Wollastonite < WFM + BaSO_4_ = WFM + CaF_2_ < WFM + Secar^®^71 < pristine WFM”.

#### 5.3.2. SEM Results

From SEM imaging, it was observed that all samples developed cracks after the Alcan test, except the sample containing both BaSO_4_ and CaF_2_, which are thought to prevent spinel formation.

(i) Pristine WFM sample

**Figure 8:**
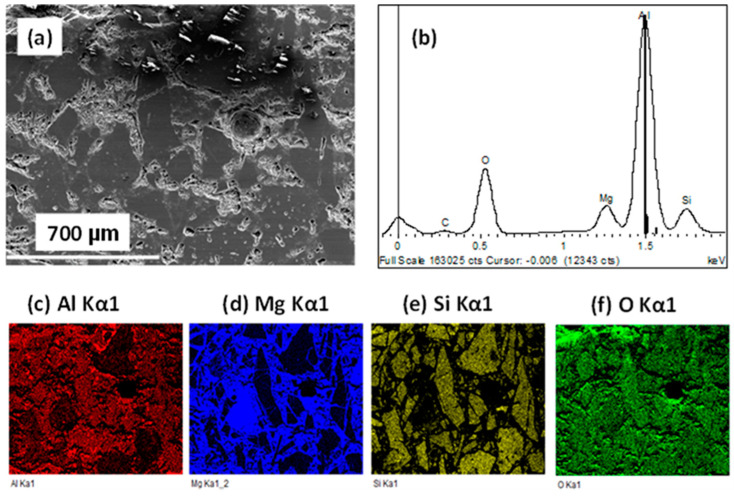
SEM imaging after Alcan test indicating (**a**) the secondary electron image of pristine WFM; (**b**) EDX spectrum of the sample; (**c**–**f**) EDX elemental mapping of Al, Mg, Si and O, respectively.

Figure 8 shows typical SEM images with EDX map scans of WFM sample after Alcan immersion test. Since XRD analysis detected the spinel phase in the sample, it can be assumed that the formation of the MgAl_2_O_4_ spinel is a strong indication of corrosion. This is because it has already been reported as the product of reaction between alumina of the mullite and the Mg of the alloy [111]. On the other hand, corundum is the product of the reaction between the SiO_2_ of mullite with Al. Although some unreacted mullite was also detected in the XRD analysis, the EDX elemental map in Figure 8 shows the formation of the spinel network, which is totally dispersed in the cross-sectional area of the sample. Therefore, pristine WFM without NWAs exhibits poor corrosion resistance when contacted with molten Al-Mg alloy.

(ii) WFM-CaF_2_-BaSO_4_ sample

**Figure 9:**
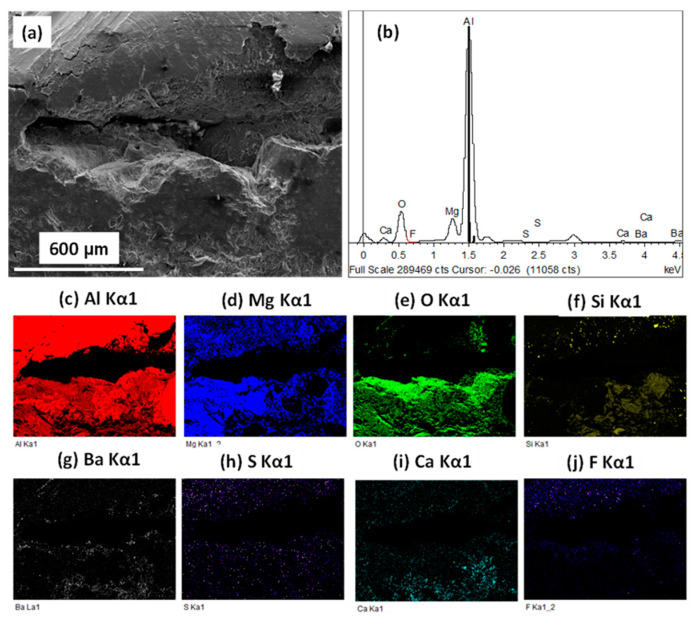
SEM imaging of WFM-CaF_2_-BaSO_4_ after Alcan test indicating (**a**) the secondary electron image of the sample; (**b**) EDX spectrum of the sample; (**c**–**j**) EDX elemental mapping of Al, Mg, O, Si, Ba, S, Ca and F, respectively.

SEM images and EDX elemental mapping of the WFM promoted with BaSO_4_ and CaF_2_ are presented in Figure 9. The XRD analysis indicated that this sample contained unique phases; namely, anorthite (CaAl_2_Si_2_O_8_), MgF_2_ and CaS (Table 5).

Since these phases inhibit the formation of corundum and consequently the production of the spinel, it is harder for molten Al to stick and react with the surface. It has been shown that the sticking efficiency or the adhesion of molten Al is much stronger on the α-Al_2_O_3_ (corundum) surfaces that are oxygen-terminated than on the Al-terminated or O-deficient surfaces [112]. This is because of the instability in O-terminated surfaces arising from their polarity, while Al-terminated surfaces are nonpolar, and therefore more stable. It is asserted that the presence of H_2_ and/or water vapor may change the Al-terminated surfaces to O-terminated ones.

We think that, at the bellyband, where atmospheric oxygen and water vapor are readily available in the furnace, the corundum surfaces are O-rich and therefore, the adhesion forces of molten Al are stronger. However, the addition of BaSO_4_ and CaF_2_ to the refractory lowers the formation of corundum and therefore the spinel. Where spinel formation is minimized as observed in the XRD patterns (Figure 6), the presence of cracks diminishes (Figure 5), and the diffusion of molten Al-Mg into the refractory, as well as the capacity to stick to the surface, are suppressed. Figure 9a is a SEM image of the WFM-CaF_2_-BaSO_4_ sample after the Alcan immersion test, and it portrays a gap between the molten Al-Mg alloy and the refractory surface. This is a clear sign of the weakening of the interfacial bonds between molten Al-Mg and the refractory surface. Figure 9b indicates the EDX spectrum of the sample, while Figure 9c–j is the EDX elemental mapping of Al, Mg, O, Si, Ba, S, Ca and F, respectively.

(iii) WFM-BaSO_4_, WFM-CaF_2_, WFM-Secar^®^71 cement and WFM-Wollatonite sample

**Figure 10:**
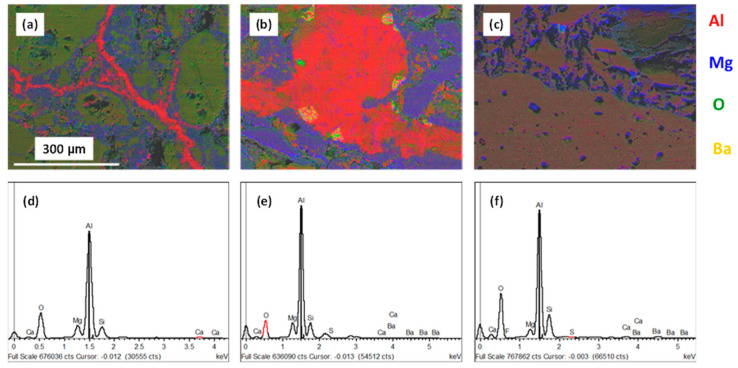
EDX elemental mapping by SEM imaging indicating the growth of cracks in (**a**) WFM+CaF_2_ and (**b**) WFM+BaSO_4_, while there are no cracks in (**c**) WFM+BaSO_4_+CaF_2_ with (**d**–**f**) showing the corresponding EDX spectrum of the samples.

Figure 10 displays SEM images and EDX map scans of WFM, containing BaSO_4_, CaF_2_ and a blend of BaSO_4_ and CaF_2_ after the Alcan immersion test. The growth of cracks and diffusion of the molten Al-Mg alloy into the refractory is evident in the EDX elemental mapping shown in Figure 10a for the sample containing CaF_2_ only, and Figure 10b for the sample containing BaSO_4_ only.

These are typical images for all the other WFM samples modified using Secar^®^71 cement and wollastonite. On the other hand, no cracks were observed in Figure 10c, which is the sample that was promoted by a combination of both BaSO_4_ and CaF_2_. The red coloration in Figure 10a,b indicates the Al-filled cracks, which are absent in Figure 10c. Figure 10d–f shows the corresponding EDX spectra of the samples. Where both BaSO_4_ and CaF_2_ were used to improve the performance of WFM, less of the spinel was evident and more anorthite was detected. Generation of anorthite has been perceived to be beneficial in the process, because it is a stable phase which does not easily react with Al-Mg alloy to form a layer of corundum. It has been observed that both CaF_2_ and anorthite are individually resistant materials when in contact with molten Al [65].

#### 5.3.3. Wettability Results

Table 6 summarizes the surface energy measurements of the samples. It was observed that pristine WFM had the highest surface energy, with a value of 22.5 mJ·m^−2^, while those of the modified samples were in the range of 20 (± 1) mJ·m^−2^. The wettability of a material is generally reduced when the overall surface energy of the solid surface is lowered [113]. From these results, it is not possible to infer a ranking for the wetting behavior at high temperature, other than that the pristine sample is significantly more prone to wetting.

## 6. Conclusions

With strong economic and environmental forces driving change in the Al industry, the Alcoa and Rio Tinto Elysis consortium has developed an innovative process to produce clean Al. The next big concern therefore is corrosion of refractories in contact with molten Al or its alloys. Two main problems associated with the corrosion of refractories include contamination of the final Al product and shortened lifespan of the refractories due to degradation. Currently, many researchers are seeking ways of improving the corrosion resistance of these refractories, and a possible solution involves the addition of non-wetting additives (NWAs) to their formulations.

In this review, some research has been conducted on corrosion, its mechanisms and potential solutions using a mullite-based (3Al_2_O_3_·2SiO_2_) refractory. A case study, which involves white fused mullite (WFM), was presented using the standard Alcan immersion test (performed at 850 °C for 96 h). However, since a high percentage of SiO_2_ is added to lower porosity, the refractories are prone to corrosion by molten Al and its alloys. As predicted by thermodynamics, the refractory aggregates start to react with molten Al at 815 °C. Even for samples with almost no open porosity (of less than 0.6%), silica or silica-containing minerals will react with molten Al metal. Reaction with silica-rich refractories leads to a volume contraction, due to the formation of corundum. The voids formed in the materials as a result of this volume decrease act as “suction pipes”, causing molten Al to penetrate the material, thus triggering corrosion above the metal line in the melting furnace. On the other hand, reactions causing volume expansions may equally generate cracks in the samples, and as such, advance further metal diffusion and reaction.

Using FactSage^TM^ thermodynamic software, Gibbs’ free energy of the corrosion reaction on aluminosilicate refractories was calculated. From the reactivity potential of pure mullite in direct contact with molten 5-wt.%Mg/Al between 850 °C and ambient temperature, the formation of corundum and spinel was confirmed. In our tests, six samples were synthesized and tested for corrosion at Pyrotek Inc., (Sherbrooke, QC, Canada). The NWAs added to WFM to improve its corrosion resistance included 2-wt.% of BaSO_4_, CaF_2_, Wollastonite, Secar^®^71, and another sample having a mixture of 1-wt.% BaSO_4_ and 1-wt.% CaF_2_.

The samples were characterized by XRD, optical microscopy, and SEM imaging, coupled with X-ray elemental mapping and surface energy measurement at room temperature. It was observed that cracks formed in the refractories where concurrent formation of spinel and corundum occurred after the Alcan immersion test. However, the sample containing a mixture of both BaSO_4_ and CaF_2_ did not generate cracks, potentially because of a combination of two reasons: (i) improved non wetting properties assessed by room temperature surface energy measurements from 22.5 mJ/m^2^ for the pristine WFM dropping to 21.1 mJ·m^−2^; and (ii) the absence of the spinel phase formation, which was not detected after the Alcan immersion test.

## Figures and Tables

**Table 1:** Gibbs free energy and enthalpy of reactions of Al industry refractories at 750 °C.

Equation Number	Reaction	∆G^o^ (kJ)	∆H^o^ (kJ)	Reference
(1)	4Al(l) + 3O_2_(g) → 2Al_2_O_3_(s)	−2708.0	−3387.0	[41]
(2)	3SiO_2_(s) + 4Al(l) → 2Al_2_O_3_(s) * + 3Si(s)	−528.0	−673.0	[64]
(3)	3Mg(l) + 4Al_2_O_3_(s) → 3MgAl_2_O_4_(s) ** + 2Al(l)	−208.0	−202.0	[65]
(4)	3Mg(l) + 1.5SiO_2_(s) → 3MgO(s) + 1.5Si(s)	−383.0	−469.0	[40]
(5)	3(3Al_2_O_3_·2SiO_2_) (s) + 8Al(l) → 13Al_2_O_3_(s) + 6Si(s)	−1033.0	−1416.0	[66]
(6)	Mg(g) + ½O_2_(g) → MgO(s)	−491.0	−609.0	[67]
(7)	2SiO_2_(s) + 2Al(l) + Mg(l) → MgAl_2_O_4_(s) + 2Si(s)	−422.0	−516.0	[59]
(8)	MgO(s) + Al_2_O_3_(s) → MgAl_2_O_4_(s)	−30.0	−230.0	[68]
(9)	2Mg(l) + SiO_2_(s) → 2MgO(s) + Si(s)	−255.0	313.0	[59]
(10)	3Mg(l) + Al_2_O_3_(s) → 3MgO(s) + 2Al(l)	−119.0	−133.0	[68]
(11)	3MgO(s) + 2Al(l) → 3Mg(l) + Al_2_O_3_(s)	+119.0	133.0	[68]
(12)	Mg(l) + ½O_2_(g) + Al_2_O_3_(s) → MgO·Al_2_O_3_(s)	−520.0	−632.0	[59]
(13)	3Mg(l) + MgAl_2_O_4_(s) → 4MgO(s) + 2Al(l)	−88.0	−109.0	[68]
(14)	2Mg(l) + Si(s) → Mg_2_Si(s)	−100	−63	[63]
(15)	BaAl_2_Si_2_O_8_(s) ^֎^ + 2⅔Al(l) → 2Si(s) + BaAl_2_O_4_(s) + 1⅓Al_2_O_3_(s)	248.0	-	[69]
(16)	8Al(l) + 3Al_6_Si_2_O_13_(s) → 13Al_2_O_3_(s) + 6Si(s)	−1040.0	−1415.0	[70]
(17)	CaO(s) + Al_2_O_3_(s) + 2SiO_2_(s) → CaAl_2_Si_2_O_8_(s) ^֎֎^	−126	−104	[66]
(18)	CaF_2_(s) + Al_2_O_3_(s) + 2SiO_2_(s) → CaAl_2_Si_2_O_8_(s) + SiF_4_(g)	−1440	−1082	[66]

* Corundum; ** Spinel; ^֎^ Celsian; ^֎֎^ Anorthite.

**Table 2:** Ingredients used to produce the plain white-fused mullite (WFM).

Ingredient	Composition	Particle Size (µm)	wt.%	Supplier
Aggregates	Mulcoa 70-80	2380	13.20	Imerys, Andersonville, IN, USA
	Mulcoa 70-20	840	18.76	Imerys, Andersonville, IN, USA
	40 white fused mullite	420	31.53	Imerys, Niagara Falls, NY, USA
Fines	Tabular Alumina-325 TA	44	4.70	Aluchem, Reading, PA, USA
	0.08 White fused mullite	80	12.42	Imerys, Niagara Falls, NY, USA
	Reactive Alumina A20SG	D50 = 3.3	8.00	Almatis, Leetsdale, PA, USA
	Reactive Alumina CTC50	D50 = 1.5	11.39	Almatis, Leetsdale, PA, USA
Liquid	Ludox 1144 (Colloidal silica) *	0.015	12.00	Nalco, Burlington, ON, Canada

* Colloidal silica contains 40-wt% solid (15 nm particles) suspended in liquid.

**Table 3:** Firing conditions.

Temperature Range (°C)	Heating Rate (°C·min^−1^)	Holding Time (h)
20–350	10	5
350–1400	15	12
1400–1400	0	5
1400–1000	10	5
1000–25	Cooling in furnace	-

**Table 4:** Mechanical properties of modified refractory samples.

Sample ID	Mechanical Strength (MPa)		Young’s Modulus (GPa)
	Flexural	Compressive	
Pristine WFM	16.30	148.46	4.89
WFM + 2-wt.% Secar^®^71	14.32	125.38	4.19
WFM + 2-wt.%BaSO_4_	13.90	153.21	4.64
WFM + 2-wt.%CaF_2_	13.10	100.36	4.19
WFM + 2-wt.%Wollastonite	12.98	165.18	4.36
WFM + 1-wt.%BaSO_4_ + 1-wt.%CaF_2_	12.88	120.97	4.39

**Table 5:** Summary of phases detected by XRD analysis in the samples after Alcan immersion test shown in Figure 6 and Figure 7.

Materials	Al_2_O_3_	SiO_2_	MgAl_2_O_4_	CaAl_2_Si_2_O_8_	Al_2_BaO_4_	MgF_2_	AlF_3_	3Al_2_O_3_·2SiO_2_	Others
WFM (Figure 7a,b)	√	X	√	X	X	–	X	–	–
WFM + BaSO_4_	√	√	√	X	√	–	X	√	MgO
WFM + CaF_2_	√	√	√	–	X	√	–	√	Mg_2_Si
WFM + BaSO_4_ + CaF_2_	√	√	–	√	–	√	–	√	CaS
WFM + Wollastonite	√	√	√	–	X	–	X	√	MgSiO_3_
WFM + Secar^®^71	√	–	√	–	X	–	X	√	Ca_3_Al_2_Si_2_

CaAl_2_Si_2_O_8_ (Anorthite); Al_2_O_3_ (Corundum), MgAl_2_O_4_ (Spinel), 3Al_2_O_3_·2SiO_2_ (Mullite), Al_2_BaO_4_ (Barium aluminate). (√): Phase detected; (X): Phase was not detected; (–): Possibility of formation.

**Table 6:** Measured surface energies of the refractories at room temperature using the Van Oss theory.

Sample	Surface Energy @ 25 °C (mJ/m^2^)
Pristine WFM	22.5
WFM-BaSO_4_	21.1
WFM-Wollastonite	19.2
WFM-CaF_2_	21.0
WFM-CaF_2_ + BaSO_4_	21.1
WFM-Secar	21.1
